# Mechanisms of Translocation of ER Chaperones to the Cell Surface and Immunomodulatory Roles in Cancer and Autoimmunity

**DOI:** 10.3389/fonc.2015.00007

**Published:** 2015-01-29

**Authors:** Valerie R. Wiersma, Marek Michalak, Trefa M. Abdullah, Edwin Bremer, Paul Eggleton

**Affiliations:** ^1^Department of Surgery, Translational Surgical Oncology, University Medical Center Groningen, University of Groningen, Groningen, Netherlands; ^2^University of Exeter Medical School, Exeter Devon, UK; ^3^Department of Biochemistry, University of Alberta, Edmonton, AB, Canada

**Keywords:** calreticulin, damage associated molecular patterns, ER stress, immunogenic cell death, post-translational modification

## Abstract

Endoplasmic reticulum (ER) chaperones (e.g., calreticulin, heat shock proteins, and isomerases) perform a multitude of functions within the ER. However, many of these chaperones can translocate to the cytosol and eventually the surface of cells, particularly during ER stress induced by e.g., drugs, UV irradiation, and microbial stimuli. Once on the cell surface or in the extracellular space, the ER chaperones can take on immunogenic characteristics, as mostly described in the context of cancer, appearing as damage-associated molecular patterns recognized by the immune system. How ER chaperones relocate to the cell surface and interact with other intracellular proteins appears to influence whether a tumor cell is targeted for cell death. The relocation of ER proteins to the cell surface can be exploited to target cancer cells for elimination by immune mechanism. Here we evaluate the evidence for the different mechanisms of ER protein translocation and binding to the cell surface and how ER protein translocation can act as a signal for cancer cells to undergo killing by immunogenic cell death and other cell death pathways. The release of chaperones can also exacerbate underlying autoimmune conditions, such as rheumatoid arthritis and multiple sclerosis, and the immunomodulatory role of extracellular chaperones as potential cancer immunotherapies requires cautious monitoring, particularly in cancer patients with underlying autoimmune disease.

## Introduction

The endoplasmic reticulum (ER) is one of many specialized organelles in the cell with diverse and apparently ever expanding functionality. When the ER was first observed in chick embryonic cells by electron microscopy, it was simply described by Porter, Claude and colleagues as one of many “submicroscopic cytoplasmic components” (1). The term “endoplasmic reticulum” was adopted by Porter and Palade because of its general morphology and intracellular location (2). Palade in his original *Science* article (3), described the ER as an “organ of complex geometry that endows it with a large surface for trapping proteins for export.” Once the subcellular fractionation of the ER organelle was possible (4), two of the major functions of the ER, namely calcium sequestration (5) and the correct assembly, folding and secretion of glycoproteins became established over the pursuing decades (6–8).

In particular, a number of proteins within the ER were discovered to be critical for the correct quality controlled folding and assembly of nascent glycoproteins – these proteins were termed chaperones and included a wide array of unrelated protein families. Chaperones are also involved in protein repair after episodes of cell stress, especially thermal shock, hence several proteins are termed “heat shock proteins (HSP)”. Some of the most plentiful luminal ER chaperones and folding enzymes in order of relative abundance are HSP47, binding immunoglobulin protein (BiP), ERP57, protein disulfide isomerase (PDI), gp96 (GRP94; HSP90), and calreticulin (9), which all fulfill unique functions required for protein assembly. For instance, PDI, a folding enzyme, assists in the correct joining of cysteine residues to create reduced disulfide bonds in nascent proteins in order to form thermodynamically stable proteins. PDI is present in millimolar quantities in the lumen of the ER of secretory cells, reflecting its importance in disulfide bond formation (10). Other proteins within the ER work in unison with isomerases to help fold, glycosylate, and post-translationally modify the majority of the 18,000 proteins that are transported to other organelles, the cell surface or beyond (11). Chaperones and folding enzymes are also involved in a number of intracellular immune functions including the formation of MHC class I and II molecules and antigen peptide loading.

During chemical or physical cell stress, the expression of chaperones are rapidly increased. Likely reasons for this rise in chaperone production are: (a) an attempt to generate correctly folded proteins to help the cell survive or, (b) to assist in shutting down the protein manufacture and aiding degradation in preparation for cell death. Another consequence of this stress response may be the relocation of chaperones to the cell surface via a number of pathways and the eventual release of chaperones into the extracellular space. On the surface, or in the extracellular space, some chaperones can signal the innate immune system to target “sick/abnormal” cells for engulfment and subsequent activation of adaptive immune responses. Indeed, the presence of chaperones on the cell surface or in the serum, is associated with disease, particularly cancers and autoimmune diseases (Table 1). Of note, chaperone proteins operating within the ER do so in an environment very different from that in other organelles or outside of cells. For example, the ER has a greater oxidizing environment with high Ca^2+^ (~1 mM) and the number and frequency of proteins is more abundant than in other organelles (12, 13). In this review, we describe the functions of ER chaperones in immunity, and discuss the different mechanisms of ER protein translocation and their possible roles in various disease pathologies.

**Table 1:** **Summary of abundant ER chaperones detected on the cell surface or in the extracellular environment and their association with various diseases**.

Protein	Localization outside ER	Potential therapeutic	Over/under expression in diseases	Reference
HSP47/serpin peptidase inhibitor clade H, member 1 (SERPINH1)	Extracellular matrix and serum	microRNA-29a (miR-29a) down regulates HSP47 and inhibits cell migration and invasion in cervical squamous cell carcinoma	HSP47 overexpressed in scirrhous carcinoma of the stomach, rheumatoid arthritis, systemic lupus erythematosus, and Sjögren’s syndrome	(14–16)
BiP/GRP78	Cell surface, nucleus	HKH40A, an 8-methoxy analog of WMC79, downregulates BiP, activates the UPR pathway and directly degrades the protein	Many cancers, especially solid tumors and musculoskeletal diseases overexpress BiP	(17–20)
ERP57	Cell surface, nucleus, cytosol, extracellular matrix, urine	Enhanced increase in cell surface ERP57 and calreticulin may enhance anthracycline-induced apoptosis	Under expression of ERP57 in breast and gastric cancer cells	(21–24)
PDI	Cell surface	Propynoic acid carbamoyl methyl amides small molecules can act as PDI inhibitors to treat ovarian cancer	PDI is upregulated in CNS cancers, lymphoma’s ovarian, lung and prostate cancer	Reviewed by (25, 26)
GRP94/gp96	Cell surface, transmembrane	GRP94 siRNA may be useful in reducing resistance of human ovarian cancer cells to chemotherapy	Upregulated in breast and ovarian cancer, lung and pancreatic cell lines	(27–30)
Calreticulin	Cell surface, extracellular, cytosol	Photofrin- and hypericin-based photodynamic therapy increases cell surface calreticulin increasing anti-tumor host responsiveness	Calreticulin is upregulated in many cancers and musculoskeletal diseases	Reviewed in (31–34)

*BiP, binding immunoglobulin protein; PDI, protein disulfide isomerase; UPR, unfolded protein response*.

## Extracellular Chaperones Can Act as DAMPs

The presence of so-called Pathogen-Associated Molecular Patterns (PAMPs) on e.g., microbes acts as a “danger signal” for the innate and adaptive immune system and helps the immune system to mount protective responses. Many intracellular host and “self” proteins that are not normally presented to the immune system similarly act as danger molecules or “alarmins” upon their release from (dying) cells. One of the most prominent of such so-called Damage Associated Molecular Patterns (DAMPs) is the high-mobility group box 1 (HMGB1) DNA binding protein. HMGB1 normally resides in the nucleus of cells, loosely bound to chromatin, but is released into the extracellular space during necrosis. This in contrast to apoptosis, where the interaction between HMGB1 and chromatin is strengthened, thus preventing the release of HMGB1 (35). Once in the extra-cellular environment, HMGB1 acts as danger signal that leads to the maturation of dendritic cells by binding to the receptor for advanced glycation end products (RAGE) and via the Toll-like receptors, TLR2 and TLR4. This subsequently triggers clonal T-cell expansion and ultimately leads to the killing of targets cells. Of note, dendritic cells also release their own HMGB1 upon activation, which amplifies their activation and is required for clonal expansion, survival, and functional polarization of naive T-cells (36). Similarly, HMGB1 is actively secreted by monocytes and macrophages upon their activation, resulting in increased HMGB1 serum levels as shown in mice (37).

Although ER chaperones are actively retained in the ER and should normally not be immunogenic, many reports have highlighted their role as DAMPs in the extracellular space. ER chaperones like calreticulin, BiP, and gp96 can activate the immune system once secreted in the extracellular space. In this respect, calreticulin was found to be the major determinant in the process of immunogenic cell death (ICD), as described in detail below (see Calreticulin Exposure Determines ICD). Similarly, tumor-secreted BiP induced antigen-specific anti-tumor responses by activating CD8 T-cells in murine cancer models (38). In addition, extracellular gp96 can also elicit tumor-specific immunity (39). Thus, ER chaperones released in the extracellular space induce (specific) immune responses. (17, 21, 40–44). The mechanism(s) by which such ER chaperones elicit immunity is not fully understood and may differ between respective chaperones. There is a substantial amount of evidence to suggest post-translational modifications of chaperones and peptide processing of chaperones changes the function and immunogenicity of at least some chaperones (see also below Retrotranslocation and Post-Translational Modification of Chaperones). For instance, in rheumatoid arthritis, citrullinated calreticulin is highly prevalent in the synovial tissue (45). This citrullinated calreticulin preferentially binds to the shared epitope (SE), a sequence motif in the β1 domain of the HLA-DR molecule that is found in 90% of rheumatoid arthritis patients, and potentiates 10,000-fold greater SE-activated signaling in innate immune cells compared to non-citrullinated calreticulin (45, 46). Furthermore, signaling via the SE was blocked by anti-calreticulin antibodies, but also by antibodies against CD91. CD91 (alpha 2-macroglobulin receptor or the low density lipoprotein-related protein) is a receptor involved in endocytosis, and has also been described to regulate the immunogenicity of other ER chaperones like gp96, HSP90, and HSP70 (47).

Due to their protein folding function, extracellular chaperones are often present in complexes with antigenic peptides, which were generated in the cells from which they were released. In order to elicit an antigen specific immune response, these chaperoned peptides needs to be re-presented by antigen presenting cells. Indeed, gp96 can be re-presented by antigen presenting cells via cell surface receptor CD91, whereby the chaperone and its bound peptide are endocytosed. The chaperone–peptide complex then enters several trafficking and processing pathways, whereupon chaperone-derived peptides are re-presented on both MHC class I and II molecules to CD8^+^ and CD4^+^ T-cells, respectively. This process allows activation of both adaptive and indirectly innate immunity against Meth A fibrosarcoma (48). Similarly, gp96 release during virally induced lytic cell death induced activation of specific T-cells when tissue supernatant was pulsed onto antigen presenting cells (49). Besides, gp96 (47, 50), heat-shock treatment of Meth A fibrosarcoma induced HSP70 expression, which did not impair proliferation or cell viability. However, these cells failed to form a tumor mass when injected in mice (51). Further, heat-shocked murine leukemia cells elicited an anti-tumor immune response and protected against tumor formation upon re-challenge due to expression of HSP60 and HSP72 (52). This immune activating response depended on the maturation of dendritic cells and activation of cytotoxic T-cells (53). In addition, the co-injection of purified HSP70 with non-immunogenic apoptotic leukemia cells potently generated anti-tumor immunity (54). Similarly, co-injection of non-immunogenic apoptotic colon or melanoma cells with calreticulin induced curative and protective T-cell immunity (55). However, extracellular calreticulin can also bind to C1q opsonized apoptotic cell debris and CD91 on monocyte/macrophages, leading to removal of apoptotic cells in a non-inflammatory manner (56). Of note, this pathway appears to be dysfunctional in some autoimmune diseases (57).

Taken together, despite their specialized functions in the ER, chaperones can be present in other cellular compartments, can be exposed on the cell surface, or may be released in the extracellular space. Once outside the cell, chaperones can act as DAMPs and activate the immune system, which may promote the clearance of infections or induce an anti-tumor immune response, but may also result in autoimmunity. The exact mechanism of the immune promoting effects of chaperones is not yet fully understood and may differ from chaperone to chaperone, but is often associated with the receptor CD91.

**Figure 1:**
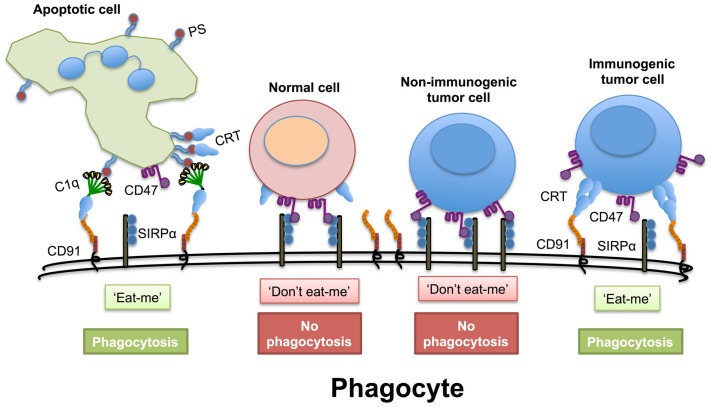
**Disruption of the “don’t eat me” signal**. Cells express the “don’t eat me” signaling molecule CD47 on their cell surface that interacts with SIRPα on phagocytes. This must be overridden when cells are preparing to die. During apoptosis, normal cells express greater amounts of phosphatidylserine (PS), which both the first component of complement (C1q) and calreticulin (CRT) can bind to directly. Extracellular calreticulin can act as a bridging molecule between C1q and CD91 on phagocytes and enhance the uptake of apoptotic cells. Even if normal cells have transient non-PS bound calreticulin on their cell surface this may not be sufficient to override the CD47− SIRPα “don’t eat me” signal. Non-immunogenic tumor cells have high levels of CD47 on their cell surface to avoid phagocytosis. However, immunogenic tumor cells have high levels expression of calreticulin on their cell surface that appears in punctate patches that can promote an “eat me” signal.

Another intriguing function of ER chaperones in the extracellular space, in particular calreticulin, is their ability to counteract “don’t eat me” signals displayed on cells (Figure 1). Healthy cells and tumor cells display the “don’t eat me” CD47 molecule. However, many types of cancer cells express higher quantities of CD47 compared to normal cells. When cells express CD47 on their cell surface it helps them avoid phagocytosis, as CD47 engages with the anti-phagocytic receptor SIRPα on phagocytic cells (58). The administration of anti-CD47 blocking antibodies enhances phagocytic uptake of tumor cells, but surprisingly not healthy cells (59). The latter finding suggests that tumor cells possess an extra signaling molecule that promotes phagocyte activity against tumor cells that is absent on healthy cells. Several authors have suggested that this overriding “eat me” signal on tumor cells is calreticulin, which cannot be substituted by other chaperones (58, 60). However, this may not be the complete picture of tumor cell recognition, as calreticulin is also expressed to varying degrees on non-apoptotic cells. Therefore, the distribution of native or post-translationally modified isoforms of calreticulin on the cell surface and its association with other co-stimulants may be necessary for efficient targeting of cells for phagocytosis. A number of co-factors identified by ourselves and others aid in surface expression of calreticulin, including ATP, Lysyl tRNA, and ERP57 (50, 61, 62). Thus, in addition to the immune activating properties shared by calreticulin with other extracellular chaperones, calreticulin is also an important player in phagocytosis by counteracting the inhibitory signaling provided by CD47.

## Appearance of Extracellular ER Chaperones and Autoantibodies in Disease States and Induction of Immunity

During disease, cells are often exposed to high levels of stress that may eventually lead to cell death. Stress and cell death may trigger release of intracellular proteins like chaperones. In line with this, extracellular calreticulin is present in the synovial fluid surrounding the joints of patients with rheumatoid arthritis (43, 63). When proteins that normally reside intracellular become exposed to the immune system, this likely induces (auto)antibody responses. Indeed, early studies demonstrated that ER chaperones are target of autoimmunity in murine models (64) and patients (57), leading to the generation of autoantibodies against a number of chaperones in serum of patients with autoimmune diseases or malignancies (17, 21, 40–44) (Table 2). Thus, ER chaperones are being released and can trigger autoantibody formation. This release occurs most likely from dead, dying, or stressed cells and may be accompanied by their post-translational modification. For instance, a number of autoimmune diseases are known to have increased cell death in the form of dysfunctional apoptosis and increased necrosis (65), leading to an array of highly concentrated chaperone proteins in membrane bound ER “blebs.” Here, these chaperones are susceptible to attack by reactive oxygen and nitrogen species, leading, e.g., to nitrosylation. Such post-translational modifications may make ER chaperones sufficiently “foreign” as to elicit an immune response. Whether the initiation of an immune response to ER chaperones is simply a reflection of a “normal” preventative autoimmune reaction that ensures removal of dying and/or damaged cells, or a precursor to autoimmune disease has been debated ever since the proposal of the “danger theory” model in 1994 (66).

**Table 2:** **The generation of anti-chaperone antibodies in autoimmune diseases and cancers**.

Disease	Anti-chaperone	Reference
**AUTOIMMUNE DISEASES**		
Autoimmune hepatitis	Anti-ERp57 IgG	(67)
Inflammatory bowel disease	Anti-calreticulin/BiP IgG	(44, 68)
Juvenile idiopathic arthritis	Anti-BiP IgG	(40)
Myasthenia gravis	Anti-GRP94 IgG	(69)
Primary biliary cirrhosis	Anti-calreticulin IgA	(70)
Rheumatoid arthritis	Anti-calreticulin/BiP/GRP94/calnexin IgG	(43, 44, 71)
SLE	Anti-calreticulin IgG/anti-PDI IgG/BiP/GRP94/calnexin	(44, 72, 73)
Systemic sclerosis	Anti-BiP/GRP94/calnexin IgG	(44)
**CANCERS**		
Colorectal carcinoma	Anti-BiP IgG	(74)
Refractory celiac disease	Anti-calreticulin IgA	(75)
Pancreatic cancer	Anti-calreticulin IgG	(76)
Melanoma	Anti-GRP94	(77)
Hepatoma	Anti-PDI IgG	(73)

**Figure 2:**
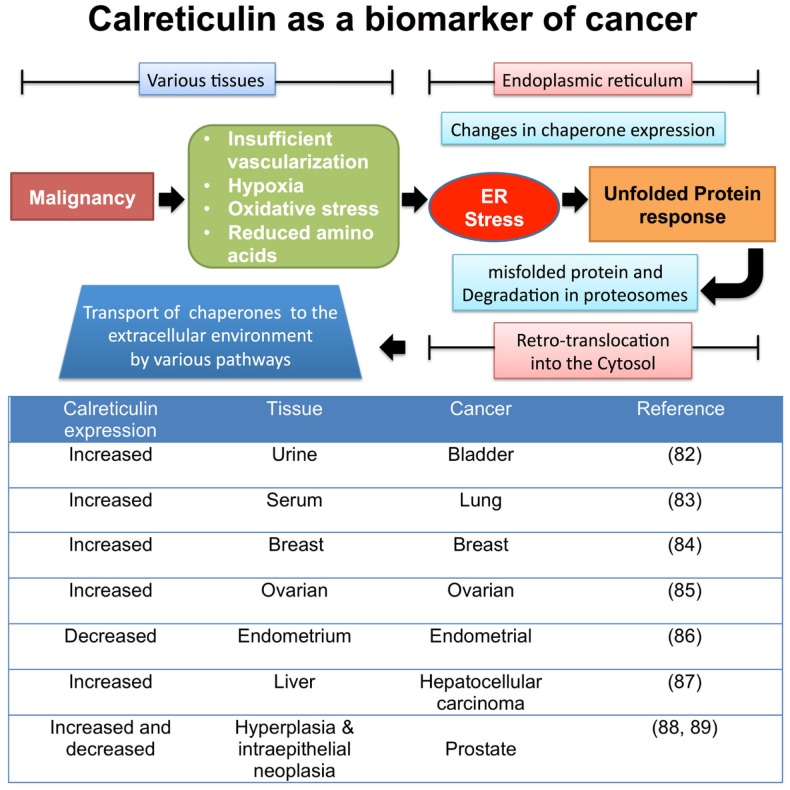
**Tumor factors that lead to changes in chaperone expression during ER stress**. Once tumors begin to proliferate in various tissues, the local microenvironment begins to become “stressed” leading to a change in metabolic and vascular demands. The ER is required to increase the rate of protein production, involving the synthesis, folding, and secretion of proteins involved in the production of tumors. This furthers stresses the ER organelle, leading to protein production errors, triggering the unfolded protein response pathway to remove incorrectly folded proteins from the ER for degradation in the cytosol via retrotranslocation to the proteasome. Some unfolded proteins are accompanied by chaperones, and these now enter the cytosol, where via a number of proposed mechanisms can leave the cell (82–89).

Of note, the overexpression of chaperones has been considered as a sign of increased malignancy, with calreticulin in particular being over-expressed in numerous tumor tissues possibly to cope with increased ER stress (Figure 2). Whilst this may thus be simply a biomarker of increased ER stress due to malignancy, some studies have suggested chaperones are engaged directly in the spread of tumors by promoting cell proliferation (78), migration (79), and metastasis (80, 81).

The production of anti-chaperone antibodies could possibly be a mechanism to suppress innate and adaptive immune responses in autoimmunity, while inadvertently neutralizing chaperone-dependent immune responses that help prevent cancer. It is known that patients with prior autoimmune disease are at a higher risk of subsequently developing certain forms of cancer (90–93). In contrast, some patients with parasitic diseases, for example, *Trypanosoma cruzi* are more resistant to developing some forms of cancer (94–96). In a number of forms of cancer anti-chaperone antibodies have been detected (see Table 2), but the clinical relevance of chaperone antibodies in the circulation of cancer patients have not been evaluated in depth. Whether anti-chaperone antibodies enhance tumor growth by blocking detection by immune cells, or are generated to protect against tumor formation are questions that remains to be addressed.

## Mechanisms of Translocation of ER Chaperones to the Cell Surface – KDEL Motifs and Receptors

**Figure 3:**
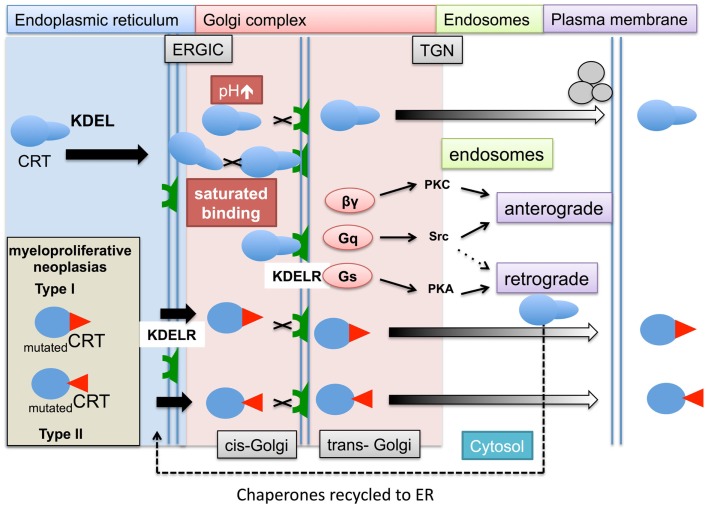
**The role of KDEL ligand and receptor in chaperone retrieval and retention within the ER and escape into the cytosol**. Within the ER, membrane bound and soluble chaperones assist in the folding (not shown) and transport of glycoproteins to the cell surface. During this process the chaperones, e.g., calreticulin (CRT) escort their cargos between the ER and Golgi complex. Upon chaperone docking to the KDEL receptors (KDELR) via their KDEL ligand, the KDELR activates a number of G proteins (βγ, Gq, and Gs) and kinases (PKC, PKA, and Src), which allows released proteins to be transported via the secretory anterograde pathway toward the plasma membrane, while chaperones are returned to the ER via a retrograde pathway. There are a number of situations in which the process of chaperones interacting with their KDEL receptors might be impaired. During ER stress induced by tumorgenesis, the ER chaperone production increases and may lead to increased saturation of the KDEL receptors with chaperones. In addition, the optimum acid pH can increase during cell stress reducing KDEL ligand/receptor interaction. In hematopoietic cells carrying Type I (52 bp deletion) and Type II (5 bp insertion) mutations in the carboxyl terminus of calreticulin, may result in lack of binding of chaperones to the KDEL receptors. This leaves the chaperones vulnerable to being trafficked by a number of secretory and alternative mechanisms into the cytosol and ultimately out of the cell.

Our own studies and those of independent researchers have focused on the release of ER-resident chaperones like calreticulin, BiP, gp96 and PDI. The ER is an industrious place of protein production and transport therefore it was argued that the chaperone proteins must be distinguished from secretory proteins to be exported in order to prevent their release via the secretory pathway. Munro and Pelham (97) identified a carboxyl terminus sequence of Lys-Asp-Glu-Leu (KDEL) on three ER-resident proteins, namely BiP, gp96, and PDI. They showed that deletion of the KDEL sequence from BiP, led to its “secretion” from mammalian cells. Subsequently many other chaperones were found to have a KDEL carboxyl terminus or a related sequence (Figure 3), including calreticulin, ERP72, and others. Chaperones armed with a KDEL sequence can safely traffic protein cargos in vesicles between the ER, Golgi complex intermediate ER-Golgi (ERGIC) complex, and Trans Golgi Network (TGN). These secretary pathway organelles and intermediates possess docking stations or KDEL receptors, which can recapture chaperones and returns them to the ER.

KDEL containing chaperones are present on the cell surface of various animal and human cells. Two decades ago gp96 was observed on mouse sarcoma (98) and *Xenopus* lymphoid cells (99). Evidence is not restricted to the transport of ER luminal chaperones. The transmembrane ER chaperone calnexin has been detected on the surface of various immature thymocyte cell lines complex with CD3 antigen, (100). At the time, it was speculated that the lack of retention of such the ER proteins was most likely during their initial formation, and that nascent ER proteins in immature hematopoietic cells may adopt a folding formation that masks their retention ligand, which is later corrected in mature thymocytes. A murine fibroblast cell line (3T6) when placed under various cell stress conditions including heat shock (43°C for 30 min), or lowering the intracellular pH with Na^+^/H^+^ transporter inhibitors or alkalizing the endosomal compartments with chloroquine, resulted in the cell surface expression of HSP47 (101). This study provided evidence that interaction of KDEL proteins binding to KDELRs is dependent upon a stable pH environment.

In humans, there are three KDEL receptor genes (KDELR1, KDELR2, and KDELR3) that encode for three types of seven transmembrane spanning KDEL receptors. KDEL receptors have a high degree of amino acid homology ~65–85%, with the KDELR3 gene producing two isoforms with even higher homology to each other. These receptors are mostly concentrated in the Golgi complex, but are also found in all of the above-described secretory organelles whereas they are absent in endosomal vesicles. The binding of chaperones requires both the KDEL sequence on the chaperone and the KDEL chaperones to be unmutated. This is exemplified by the recent discovery that patients with myeloproliferative neoplasms (MPNs) that did not have a janus kinase 2 (JAK2) mutation (a mutation occurring in the vast majority of patients) are characterized by somatic mutations in their calreticulin gene (102). Such mutations lead to release of calreticulin by megakaryocytes, possibly into the bone marrow (103). Interestingly, many of the mutations are found in the carboxyl terminus of the protein leading to changes in peptide structure. This region of calreticulin has a low affinity binding site for calcium and contains the KDEL sequence that is believed to be important in retaining the protein within the ER (104). Mutated forms of calreticulin identified in MPN lack KDEL raising the possibility that some mutated calreticulin isoforms may not be retained in the lumen of the ER by KDEL receptors, whilst other are retain in the ER despite lacking a KDEL sequence (personal communication – Prof Tony Green).

The above may account for some of the extracellular calreticulin, but does not fully explain why extracellular and cell membrane bound calreticulin are observed in other forms of cancers or in autoimmune patients (see Figure 3 and Table 1). The notion that KDEL receptors “retain” chaperones has changed over a number of years and it is now believed KDEL receptors act more as retrieval systems shepherding chaperones between the ER and Golgi complex during cell stress via retrograde (104) and allowing their protein cargoes to move toward the plasma membrane via anterograde (105) transport pathways. If these pathways are impaired, chaperones could accumulate in the cytosol in endosomal vesicles. Moreover when KDEL receptors become saturated with chaperones, non-bound chaperones may escape the retrograde retrieval system and fail to return to the ER. Certain chaperones have additional retention mechanisms. The enzyme aminoacyl-tRNA synthetase (AIMP1) enhances the dimerization of gp96 and aids greater retention of gp96 by the KDEL-1 receptor; suggesting different ER chaperones rely on different regulatory retention mechanisms (106).

**Figure 4:**
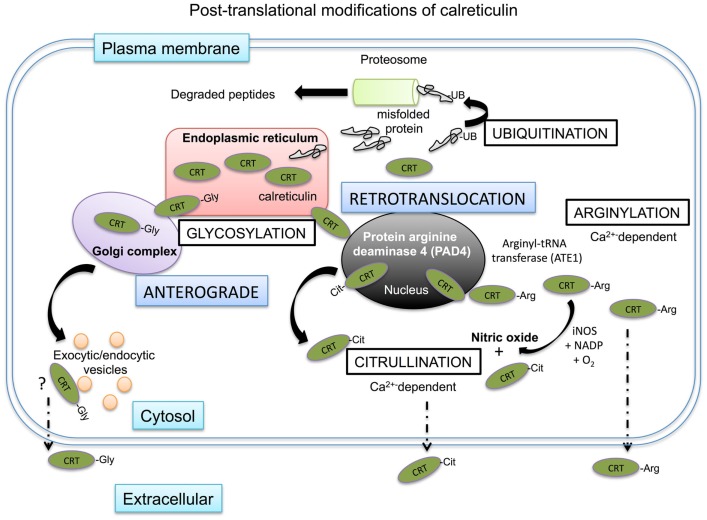
**Intracellular post-translational modifications of calreticulin**. Mis-folded proteins directly leave the ER and are ubiquitinated in the cytosol before degradation in the proteasome. Calreticulin has been shown to be transported to the cytosol possibly via the nucleus. Within the nucleus, calreticulin is exposed to protein arginase deaminase 4 (PAD4) where it may be citrullinated before being shuttled to the cytosol in association with nuclear export proteins. There is no evidence of calreticulin being ubiquitinated in the cytosol, but it does encounter arginyl-tRNA transferases, which can arginylate the protein. The addition of arginine on the protein can be further citrullinated in the cytosol in the presence of iNOS as a byproduct of nitric oxide production from the conversion of arginine to citrulline. Both citrullinated and arginylated isoforms of calreticulin have been found outside of the cell, where they exert specific biological functions. Artificial glycosylation of calreticulin leads to the secretion of calreticulin out of the cell via the secretory pathway and glycosylated isoforms of calreticulin have been observed in human myeloid cells.

A large number of mutations have also been identified in the KDEL receptor but many of these do not affect the intracellular location or KDEL binding capacity of KDEL receptors. However, retrograde transport of the KDEL containing proteins is dependent on a presence of a single aspartic acid residue in the seventh membrane-spanning region, which may be important for conformational changes and intermolecular interaction in the membrane bilayer of KDEL receptor possessing vesicles (107). The binding of KDEL ligands to the KDEL receptors leads to activation of a number of specific kinase signaling pathways, specifically activation of G-proteins (108). This triggers a series of signaling pathways (109) that can aid the return of chaperones back to the Golgi complex and ER retrograde pathways or possibly transport them toward the plasma membrane by anterograde pathways in endosomal compartments (Figure 4).

## Retrotranslocation and Post-Translational Modification of Chaperones

Many of the chaperones of the heat shock protein family are normally resident in the cytosol (47). However, other chaperones such as calreticulin are typically retained in the ER, but have also been identified in the cytosol after having somehow escaped the retrograde retention pathway between the ER and Golgi complex (Figure 4). The expression of ER chaperones on the cell surface or extracellular environment could be explained if chaperones can be demonstrated to reach the cell surface via the anterograde type secretory pathways. In the case of calreticulin, its normal physiological isoform cannot enter the secretory pathway as it is non-glycosylated. However, Panaretakis and colleagues created a glycosylated form of calreticulin that trafficked to the cell surface in an anterograde manner via the Golgi complex/actin mediated exocytic vesicle secretory pathway in murine colon cancer cell line CT26 (110). Further, a naturally glycosylated form of calreticulin has been observed in the myeloid tumor cell line HL60 (111). Therefore, in certain settings glycosylation of calreticulin may occur and may trigger secretion into the extracellular space. Such glycosylation may occur on surface exposed asparagine peptides in the P-domain of the protein that can, at least artificially, be N-glycosylated.

There is also evidence to suggest that ER chaperones can leave the ER via a retrotranslocation pathway, particularly under stress conditions (112). Mis-folded proteins retrotranslocate into the cytosol and are commonly post-translationally modified by a process of ubiquitylation. In brief, ubiquitin binds to lysines on the protein, which act as a proteasomal degradation signal for the protein (113). Afshar and colleagues, used digitonin to specifically permeabilize only the outer cell membrane of mammalian cells, while leaving the membranes of intracellular organelles intact (114). Using this strategy they recovered ~14% of total calreticulin, whilst other chaperones such as PDI and gp96 were retained in the ER. Of note, the recovered calreticulin was not ubiquitinated, suggesting that calreticulin passed into the cytosol through an ubiquitin- and proteasome-independent retrotranslocation pathway. In a series of deletion experiments, they showed that the C domain of calreticulin mediated this retrotranslocation. Reversely, insertion of the C-domain of calreticulin in PDI allowed this chaperone to retrotranslocate to the cytosol. There is some evidence to suggest that such retrotranslocation of calreticulin from the ER to the cytosol occurs via the nucleus, where it may interact with proteins with nuclear export signals and exit the nucleus in complex with nuclear proteins (115).

Whether this cytosolic calreticulin is the source of plasma-membrane calreticulin is not known for certain. However, calreticulin on the cell membrane has been found to be arginylated (116). Protein arginylation is catalyzed by a cytosolic-based enzyme, arginyl-tRNA protein transferase (ATE1). Under cell stress conditions, ATE1 can promote the linkage of arginine to N-terminal amino groups, but also to mid-chain side groups of aspartate and glutamic acid (117, 118). Such arginylated isoforms of calreticulin have been found in the cytosol associated with stress granules, but are not found in the ER (119). Once on the cell surface, arginylated calreticulin can influence cell survival, with exogenously applied arginylated calreticulin increasing cellular apoptosis and overcoming resistance to apoptosis (116). Of note, this may not be the case for other isoforms of calreticulin detected outside the cell. Interestingly in cells lacking ATE1, no calreticulin could be detected on the cell surface, suggesting that arginylation of calreticulin is a requisite for surface exposure. As mentioned earlier, another isoform of calreticulin exists in the form of citrullinated calreticulin, which was found to modulate immune function in rheumatoid arthritis patients (120, 121).

## Calreticulin, Nitric Oxide, and Inhibition of Flipases

Many chaperones and HSP in the cytosol of cells are detected on the cell surface, but very little is known as how these get out of cells via non-ERGIC pathways. Despite this, for many years some of these proteins, especially the HSP70 and HSP90 families of proteins have been known to play a number of extracellular roles in infections, autoimmune disease, and tumor-specific recognition (122). Some chaperones present in the cytosol may associate with the phospholipids facing the lumen of the cell. Heat shock protein chaperones are known to be in close proximity to the plasma membranes and assist in the translocation of proteins across the membrane for export out of the cell. In artificial lipid bilayers, HSP have been demonstrated to create ATP-dependent transmembrane ion channels (123). We showed calreticulin binds in a Ca^2+^-dependent manner directly to phosphatidylserine (PS) (112). Normally 80% of PS is located on the inner leaflet with only 20% of PS on the outer surface of healthy cells. The polar head of PS was shown to bind to CRT with high affinity (*K_D_* = 1.5 × 10^−5^ M) (124). We observed the interaction of calreticulin occurred in punctate regions of the membranes and in a further study demonstrated the calreticulin was associated with lipid rafts (as demonstrated by incorporation of cholera toxin B) in association with ERp57 (61). Whether these chaperones associated with lipid rafts can leave the cells through dimerizing and clustering in rafts that bud from the cell is unknown.

As discussed above, citrullinated calreticulin binding to the SE on the surface of effector cells can lead to NO production in opposite cells. NO production is a cell stress signal that can deplete ER Ca^2+^ and lead to overexpression of calreticulin (125). The overexpression of calreticulin may result in the protein leaving the ER by the mechanisms discussed above. The KDEL retention receptors may also become saturated preventing its retention in the ERGIC complex. The increased calreticulin further promotes intracellular NO production (126). Cytosolic calreticulin has the ability to bind to PS on the inner leaflet in a Ca^2+^ -dependent manner in close proximity to the flipase, aminophospholipid translocase (APLT). In an environment of increase NO activity, the SH groups of APLT are susceptible to transnitrosylation/oxidation, this leads to the inhibition of APLT to retain PS on the inner leaflet of the plasma membrane. Our own experiments demonstrated that Jurkat T-cells exposed to S-nitroso-l-cysteine-ethyl-ester, an intracellular NO donor and inhibitor of APLT results in PS and calreticulin externalization together in an S-nitrosothiol-dependent and caspase-independent manner (112). Other forms of cell stress also appear to promote surface expression of chaperones that can be exploited to tumor eradication as discussed below.

## Calreticulin Exposure Determines Immunogenic Cell Death

The potential pro-immunogenic role of chaperones gained prominence by the discovery that cell surface exposure of calreticulin determines the immunogenicity of cancer cell death. This so-called ICD is induced by certain chemotherapeutics, e.g., anthracycins, or irradiation, and hinges on the rapid pre-apoptotic translocation of calreticulin to the cell surface (55). Such surface-exposed calreticulin induces the uptake of dying cancer cells by CD11c-positive myeloid dendritic cells, leading to tumor antigen presentation to T-cells and concomitant clonal T-cell expansion. Injection of calreticulin-exposing dying tumor cells prevented tumor growth upon re-challenge with viable tumor cells. Selective knock-down of calreticulin reduced the phagocytic uptake of anthracyclin treated cells by dendritic cells and abolished T-cell-mediated elimination of the tumor. Analogously, apoptotic human bladder cancer cells and murine colon cancer cells treated with the photodynamic therapeutic hypericin exposed calreticulin on their membrane. Again, surface calreticulin induced maturation of human immature dendritic cells, and elicited an anti-tumor immune response in mice (50). Of note, non-immunogenic cytotoxic treatment of cancer cells was converted to immunogenic by co-treatment with recombinant calreticulin (55), highlighting the pivotal role of calreticulin in ICD.

In addition to calreticulin exposure, late apoptotic or necrotic release of HMGB-1 from dying cells, and subsequent binding to TLR-4 on dendritic cells was necessary to obtain optimal antigen presentation of chemotherapy or radiotherapy treated cancer cells (127). Indeed, dendritic cells lacking TLR-4 or its downstream adaptor molecule Myd88 could not present antigen from dying tumor cells and did not elicit a T-cell mediated anti-cancer immune response in mice (127). Further, knock-down of HMGB-1 inhibited the potential of irradiated tumor cells to stimulate dendritic cells. In addition to the role of HMGB1 in ICD, it was found that upon hypericin treatment of bladder cancer cells or upon oxaliplatin or doxyrubicin treatment high levels of ATP were secreted, which like calreticulin also preceded apoptotic PS exposure (50). Inhibition of ATP abolished the inflammatory response (128).

Based on the above, there is a cascade of events that determines the immunogenicity of cell death. Here, calreticulin is translocated to the cell membrane during early (pre-apoptotic) stages of dying tumor cells, which facilitates efficient uptake by dendritic cells. In addition, the release of ATP during early apoptotic stages is essential to mount an immune response. Further, HMGB-1 release at late apoptotic stages is required for efficient antigen presentation by dendritic cells to T-cells. Of note, whereas capsaicin treatment induced pre-apoptotic calreticulin exposure and ATP release, HSP90 and HSP70 release occurred (129). Similarly, hypericin treated cancer cells actively exposed calreticulin, with no detectable levels of HSP90, calnexin, or BiP. However, at later (late apoptotic) stages, certain levels of extracellular calreticulin, HSP70 and HSP90 were detected, as a result of passive extracellular release (31). Thus, calreticulin exposure is required to induce ICD, although several additional stimuli contribute to an efficient immune response.

## Translocation of Calreticulin to the Cell Surface during Cancer Therapy

The exact translocation pathway of calreticulin during ICD is not known. In certain cases, the chaperone ERp57 was found to steer calreticulin translocation, specifically upon anthracyclin treatment (21, 62). ERp57 and calreticulin extracellular expression levels correlated and also co-translocated to the surface of mitoxantrone treated tumor cells. Further, calreticulin and ERp57 were needed for each others translocation in mitoxantrone and radiation treated cells, as calreticulin knock-outs failed to expose both calreticulin and ERp57 to the cell surface and *vice versa* (21, 62). In contrast, the interaction between ERp57 and calreticulin was not required to induce calreticulin cell surface exposure in thapsigargin treated cells (130). Here, mouse embryonic fibroblasts (MEFs) that expressed a mutated form of calreticulin that was unable to bind ERp57, had equal amounts of cell surface calreticulin compared to wildtype MEFs during thapsigargin treatment. Similarly, the translocation of calreticulin upon hypericin photodynamic therapy was not accompanied by co-translocation of ERp57 (31, 50). However, both mitoxantrone and hypericin mediated translocation of calreticulin was blocked by Brefeldin A, an inhibitor of anterograde protein transport from the ER to the Golgi apparatus (50, 110). In addition, extracellular calreticulin but not ERp57 was required to induce phagocytosis and subsequent induction of anti-tumor immune responses (31, 62). Thus, the mechanism and routing of calreticulin to the cell surface seems to be dependent on the ICD-inducing compound, and likely also cell type dependent.

## Translocation of ER Chaperones Requires Activation of the ER Stress Response

The exposure of tumor cells to anthracycline antibiotics such as doxorubicin, mitoxantrone (55) or physical treatments such as photodynamic therapy with hypericin (32) commonly induce ER cell stress. This ER stress response appears to be an obligatory step in inducing extracellular expression of ER chaperones. In contrast, nuclear damage or signaling is not a requisite, as enucleated cells exposed calreticulin on their surface to a similar degree as observed for normal cells upon anthracyclin therapy (21).

The ER stress response via PERK and eIF2α was found to be involved in the translocation of calreticulin to the cell surface during ICD. When PERK phosphorylates eIF2α, translation initiation is halted, resulting in reduced protein synthesis. In mitoxantrone treated CT26 colon cancer cells, the translocation of calreticulin and ERp57 was accompanied by phosphorylation of PERK and its substrate eIF2α (21). Similarly, hypericin mediated photodynamic therapy induced eIF2α phosphorylation and PERK activation (50). When CT26 cells were depleted for PERK or when a non-phosphorylatable form of eIF2α was expressed, this completely abolished calreticulin/ERp57 exposure, whereas it did not affect the sensitivity toward anthracyclin induced cell death. In contrast, eIF2α was not required for hypericin induced calreticulin exposure, but solely relied on PERK activation (50). This discrepancy might rely on the pronounced localization into the ER of hypericin, whereby sufficient ER stress might already be induced upon photodynamic disruption of the organelle. In line with this, the photodynamic therapeutic photofrin, which has a less pronounced ER localization, was not able to induce calreticulin exposure (50). However, also spontaneous release of calreticulin from acute myeloid leukemia (AML) blast was associated with eIF2α hyperphosphorylation (131). Furthermore, the disruption of the PP1/GADD34 complex, a complex that is involved in the dephosphorylation of eIF2α was already sufficient to induce calreticulin exposure (55, 132). Thus, the induction of an ER stress response is required to induce extracellular calreticulin exposure, which might be induced via various pathways, depending on the therapeutic.

In addition to ER stress, the formation of reactive oxygen species (ROS) and reduction of ER Ca^2+^ levels may favor cell membrane surface exposure of calreticulin. Indeed, most therapies that can induce ICD also induce ROS formation. When CT26 cells, treated with anthracylines or radiation therapy, were incubated with ROS scavengers (N-Acetyl cysteine, glutathion ethyl ester) this prevented apoptosis as well as calreticulin exposure (110). Similarly, the presence of the ^1^O_2_ quencher l-histidine decreased calreticulin translocation in hypericin treated bladder cancer cells (50). However, the presence of redox stress alone does not suffice to translocate calreticulin, as cisplatin treated osteosarcoma cells were unable to expose calreticulin, although significant levels of apoptosis, mitochondrial damage, and ATP release were induced (133). This lack in calreticulin exposure was associated with inefficient induction of the ER stress response as eIF2α was only minimally phosphorylated upon cisplatin treatment. Of note, thapsigargin treatment alone was also inefficient for induction of calreticulin exposure, although it did phosphorylate eIF2α (110, 133). Interestingly, when cisplatin and thapsigargin therapy were combined, this restored the ER stress response and induced calreticulin exposure, which was sufficient to induce an immune response in mice (133). Of note, thapsigargin is an inhibitor of SERCA pumps, whereby the ER Ca^2+^ levels decrease, which might also contribute to ER stress. Indeed, levels of cell membrane expressed calreticulin were enhanced in thapsigargin treated neuroblastoma cells, which were genetically manipulated to have reduced Ca^2+^ levels in the ER (134). Of note, in addition to the ER stress response, a specific apoptotic response is also required in some cases, whereas it is not necessary in others. In this respect, caspase-8 activation was needed to induce calreticulin/ERp57 translocation in mitoxantrone treated CT26 cells ore MEFs, as cells depleted for caspase-8 lost their ability to translocate calreticulin/ERp57 (110). In contrast, inhibition of caspase-8 activity did not affect hypericin induced calreticulin exposure (50). Thus, ER stress and ROS production are both required for calreticulin translocation, whereas additional stimuli, i.e., caspase activation or ER Ca^2+^ depletion, are essential depending on therapeutic strategy of cell type.

## Potential Role of Extracellular ER Chaperones as Therapeutics in Cancer Therapy: Evidence for ICD in Clinical Settings

Most of the work on ICD has been performed in animal studies or *in vitro*. However, there are some studies on the existence of ICD in the clinic. For instance, a combination of heat shock/γ-ray/UV-radiation therapy was used to induce cell death in primary indolent non-Hodgkin’s lymphoma cells, which were *ex vivo* loaded on autologous dendritic cells, for vaccination strategies (135). Here, 6 out of 18 patients showed clinical and immunologic responses. Of note, the levels of calreticulin and HSP90 exposure were significantly higher in heat shock/γ-ray/UV-ray treated tumor cells from responders compared to non-responders. In line with this, clinical responders showed higher amounts of circulating antibodies against HSP90 and calreticulin after vaccination. In contrast, there was no difference in the amount of cell death or HSP70 or HMGB-1 release between tumor cells from responders and non-responders. Similarly, there was no difference in the expression of HLA class I and II. As a consequence, NK-cell maturation was increased, which directly correlated with the levels of calreticulin and HSP90 expression. In another study, the expression of cell surface calreticulin was found on AML blasts, although this was regardless of chemotherapy (131). In addition, the *in vivo* treatment of patients with anthracylines did not enhance calreticulin exposure on malignant blasts and did not alter the serum calreticulin levels. However, the presence of calreticulin on the cell surface of malignant AML blasts did associate with enhanced immune responses, since T-cells from calreticulin-positive patients produced IFNγ upon interaction with autologues dendritic cells, whereas T-cells from calreticulin-negative patients failed to respond upon this trigger. However, the overall survival of these AML patients did not correlate with calreticulin levels. The capacity of clinical drugs to induce ICD was also tested on primary patient derived ovarian and prostate cancer cells. Exposure to anthracylines was sufficient to induce translocation of calreticulin, HSP70 and HSP90 to the cell surface, and HMGB-1 release at later time point (136), but the clinical implications of ICD in these cancer types warrants further analysis. In addition to the well known ICD inducers (i.e., anthracyclins and radiation), cardiac glycosides were recently also recognized as inducers of ICD, also eliciting anti-cancer immune responses in mice (137). Using retrospective clinical analysis of human carcinoma patients, it was found that the administration of the cardiac glycoside digoxin during chemotherapy improved overall survival of patients with colorectal, breast or head, and neck cancer. However, it should be noticed that this positive effect was only observed in patients treated with chemotherapeutics considered as non-immunogenic. Indeed, the addition of digoxin failed to affect overall survival of patients that received anthracyclin therapy.

Taken together, in clinical settings, calreticulin and associated chaperones can be exposed on tumor cells or in serum from patients. However, the induction of immune responses and benefit in terms of survival are not as straightforward as postulated in animal studies. Thus, many challenges remain in terms of identifying the essential set of signal requisites for induction of ICD in order to achieve efficient immune responses upon ICD in patients.

## Challenges for the Therapeutic Implication of ICD

From the above, it appears that the induction of ICD and accompanied calreticulin exposure on tumor cells is a promising strategy to obtain curative cancer therapies in patients. However, there are several challenges that remain to be addressed. First, the induction of calreticulin exposure by anthracycline therapy seems to be hampered *in vivo* and shows a high variability between patients (131, 138). Although, calreticulin was found on malignant blast from AML patients, this was independent of therapy and caused by spontaneous release (131). Similarly, apoptotic AML cells, which died spontaneously or as a result of cytotoxic drugs in *ex vivo* assays, showed calreticulin exposure and release of HSP70 and HSP90. However, there was a wide variation in the levels between different patients, which depended on individual patient characteristics, rather than the cell death inducing therapeutics (138). Thus, ways of reliably and uniformly inducing calreticulin exposure in cancer patients will have to be identified.

Secondly, induction of ICD by a certain chemotherapeutic appears to be cell type and perhaps context-dependent. For instance, thapsigargin was found to induce an ER stress response in CT26 colon cancer cells, but failed to stimulate cellular calreticulin/ERp57 exposure (110). In contrast, thapsigargin induced both ER stress and calreticulin release in neuroblastoma and MEFs (139). In the case of the former, surface exposure of calreticulin was strongly enhanced when Ca^2+^ levels in the ER lumen were depleted (134). Also in primary cells isolated from ovarian and prostate cancer patients, anthracyclines were able to induce calreticulin exposure and release of HSP70 and HSP90, whereas there was completely no induction of ICD upon UV-radiation (136). Therefore, optimal treatment strategies need to be evaluated for each cancer type with special focus on combining different therapies to optimize induction of key immunogenic molecules. Indeed, in non-Hodgkin lymphoma cell lines (NHL), the combination of heat shock, γ-ray, and UVC-ray therapy induced higher amounts of calreticulin and HSP90 exposure, and HMGB-1 and ATP release than each single treatment (135).

Of note, many of the cytotoxic agents that in pre-clinical models of ICD elicit pre-apoptotic calreticulin exposure, such as doxorubicin, can induce severe myelosuppression and leukopenia. This toxicity may negatively affect the pro-immunogenic effect of extracellular calreticulin in patients by deleting requisite immune components of the ICD pathway. Indeed, although calreticulin-dependent ICD has been described for various cytotoxic agents in pre-clinical settings these typically have not translated into reports on effective anti-cancer immunity upon treatment of patients. In this respect, the identification of optimally immunogenic treatments with minimum toxicity toward critical immune cells seems warranted, e.g., in further combination with therapeutics that selectively target negative immunoregulatory cells such as myeloid-derived suppressor cells and regulatory T-cells.

Finally, as already discussed above, the calreticulin “eat me” signaling is counterbalanced by the “don’t eat me” signaling via CD47. For high CD47-expressing cancer it may therefore be beneficial to include CD47-blocking therapeutics in order to optimize therapeutic efficacy. In this respect, it is interesting to mention that the only study in which clinical responses to tumor expressed calreticulin was found, has been described in NHL patients (135). These NHL patients typically also show strong overexpression of CD47 (59).

## Conclusion

Chaperone molecules play a number of specific roles related to protein processing within the cell. However, new knowledge indicates that a select number of chaperones in the extracellular environment can play a role in both innate and adaptive immunity that may be useful in the treatment of tumors. In contrast, the release of potent immunogenic-stimulating molecules may have a detrimental role in some autoimmune diseases. Therefore, it is crucial to understand how various post-translational modified forms of chaperones are release from cells under resting and stressed conditions and how the released chaperones exert their immune-promoting responses. Clearly, there are several ways in which these chaperone proteins can be released from cells other than through the process of passive necrosis. Their complex interactions with the immune system, especially chaperone–immune cell signaling pathways and receptors interactions requires further studies to help understand their role of potential therapeutics to treat cancers and in their ability to induce inflammation in autoimmune disease.

## Conflict of Interest Statement

The authors declare that the research was conducted in the absence of any commercial or financial relationships that could be construed as a potential conflict of interest.

## References

[1] Porter KR Claude A Fullam EF. A study of tissue culture cells by electron microscopy: methods and preliminary observations. J Exp Med (1945) 81:233–46. 10.1084/jem.81.3.233 19871454 PMC2135493

[2] Palade GE Porter KR. Studies on the endoplasmic reticulum. I. Its identification in cells in situ. J Exp Med (1954) 100:641–56. 10.1084/jem.100.6.641 13211920 PMC2136401

[3] Palade G. Intracellular aspects of the process of protein synthesis. Science (1975) 189:347–58. 10.1126/science.1096303 1096303

[4] Palade GE Siekevitz P. Liver microsomes; an integrated morphological and biochemical study. J Biophys Biochem Cytol (1956) 2:171–200. 10.1083/jcb.2.6.671 13319380 PMC2223971

[5] Mazzarello P Calligaro A Vannini V Muscatello U. The sarcoplasmic reticulum: its discovery and rediscovery. Nat Rev Mol Cell Biol (2003) 4:69–74. 10.1038/nrm1003 12511870

[6] Krieg UC Johnson AE Walter P. Protein translocation across the endoplasmic reticulum membrane: identification by photocross-linking of a 39-kD integral membrane glycoprotein as part of a putative translocation tunnel. J Cell Biol (1989) 109:2033–43. 10.1083/jcb.109.5.2033 2808520 PMC2115841

[7] Deshaies RJ Schekman R. A yeast mutant defective at an early stage in import of secretory protein precursors into the endoplasmic reticulum. J Cell Biol (1987) 105:633–45. 10.1083/jcb.105.2.633 3305520 PMC2114772

[8] Caro LG Palade GE. Protein synthesis, storage, and discharge in the pancreatic exocrine cell. An autoradiographic study. J Cell Biol (1964) 20:473–95. 10.1083/jcb.20.3.473 14128049 PMC2106415

[9] Schwanhausser B Busse D Li N Dittmar G Schuchhardt J Wolf J Global quantification of mammalian gene expression control. Nature (2011) 473:337–42. 10.1038/nature10098 21593866

[10] Freedman RB Protein disulfide isomerase: multiple roles in the modification of nascent secretory proteins. Cell (1989) 57:1069–72 10.1016/0092-8674(89)90043-3 2544299

[11] Schachter H The subcellular sites of glycosylation. Biochemical Society Symposium London: Publisher Portland Press (1974). p. 57–71. 4620386

[12] Gidalevitz T Stevens F Argon Y. Orchestration of secretory protein folding by ER chaperones. Biochim Biophys Acta (2013) 1833:2410–24. 10.1016/j.bbamcr.2013.03.007 23507200 PMC3729627

[13] Montero M Brini M Marsault R Alvarez J Sitia R Pozzan T Monitoring dynamic changes in free Ca2+ concentration in the endoplasmic reticulum of intact cells. EMBO J (1995) 14:5467–75. 8521803 10.1002/j.1460-2075.1995.tb00233.x PMC394660

[14] Hirai K Kikuchi S Kurita A Ohashi S Adachi E Matsuoka Y Immunohistochemical distribution of heat shock protein 47 (HSP47) in scirrhous carcinoma of the stomach. Anticancer Res (2006) 26:71–8. 16475681

[15] Yokota S Kubota H Matsuoka Y Naitoh M Hirata D Minota S Prevalence of HSP47 antigen and autoantibodies to HSP47 in the sera of patients with mixed connective tissue disease. Biochem Biophys Res Commun (2003) 303:413–8. 10.1016/S0006-291X(03)00352-8 12659832

[16] Yamamoto N Kinoshita T Nohata N Yoshino H Itesako T Fujimura L Tumor-suppressive microRNA-29a inhibits cancer cell migration and invasion via targeting HSP47 in cervical squamous cell carcinoma. Int J Oncol (2013) 43:1855–63. 10.3892/ijo.2013.2145 24141696 PMC3834344

[17] Zhang Y Liu R Ni M Gill P Lee AS. Cell surface relocalization of the endoplasmic reticulum chaperone and unfolded protein response regulator GRP78/BiP. J Biol Chem (2010) 285:15065–75. 10.1074/jbc.M109.087445 20208072 PMC2865300

[18] Li J Lee AS. Stress induction of GRP78/BiP and its role in cancer. Curr Mol Med (2006) 6:45–54. 10.2174/156652406775574523 16472112

[19] Panayi GS Corrigall VM. BiP regulates autoimmune inflammation and tissue damage. Autoimmun Rev (2006) 5:140–2. 10.1016/j.autrev.2005.08.006 16431346

[20] Kosakowska-Cholody T Lin J Srideshikan SM Scheffer L Tarasova NI Acharya JK. HKH40A downregulates GRP78/BiP expression in cancer cells. Cell Death Dis (2014) 5:e1240. 10.1038/cddis.2014.203 24853418 PMC4047900

[21] Panaretakis T Joza N Modjtahedi N Tesniere A Vitale I Durchschlag M The co-translocation of ERp57 and calreticulin determines the immunogenicity of cell death. Cell Death Differ (2008) 15:1499–509. 10.1038/cdd.2008.67 18464797

[22] Dihazi H Dihazi GH Bibi A Eltoweissy M Mueller CA Asif AR Secretion of ERP57 is important for extracellular matrix accumulation and progression of renal fibrosis, and is an early sign of disease onset. J Cell Sci (2013) 126:3649–63. 10.1242/jcs.125088 23781031

[23] Gaucci E Altieri F Turano C Chichiarelli S. The protein ERp57 contributes to EGF receptor signaling and internalization in MDA-MB-468 breast cancer cells. J Cell Biochem (2013) 114:2461–70. 10.1002/jcb.24590 23696074

[24] Leys CM Nomura S LaFleur BJ Ferrone S Kaminishi M Montgomery E Expression and prognostic significance of prothymosin-alpha and ERp57 in human gastric cancer. Surgery (2007) 141:41–50. 10.1016/j.surg.2006.05.009 17188166

[25] Xu S Sankar S Neamati N. Protein disulfide isomerase: a promising target for cancer therapy. Drug Discov Today (2014) 19:222–40. 10.1016/j.drudis.2013.10.017 24184531

[26] Xu S Butkevich AN Yamada R Zhou Y Debnath B Duncan R Discovery of an orally active small-molecule irreversible inhibitor of protein disulfide isomerase for ovarian cancer treatment. Proc Natl Acad Sci U S A (2012) 109:16348–53. 10.1073/pnas.1205226109 22988091 PMC3479552

[27] Dejeans N Glorieux C Guenin S Beck R Sid B Rousseau R Overexpression of GRP94 in breast cancer cells resistant to oxidative stress promotes high levels of cancer cell proliferation and migration: implications for tumor recurrence. Free Radic Biol Med (2012) 52:993–1002. 10.1016/j.freeradbiomed.2011.12.019 22245095

[28] Zhang L Wang S Wangtao Y Wang J Wang L Jiang S Upregulation of GRP78 and GRP94 and its function in chemotherapy resistance to VP-16 in human lung cancer cell line SK-MES-1. Cancer Invest (2009) 27:453–8. 10.1080/07357900802527239 19212831

[29] Pan Z Erkan M Streit S Friess H Kleeff J. Silencing of GRP94 expression promotes apoptosis in pancreatic cancer cells. Int J Oncol (2009) 35:823–8. 10.3892/ijo_00000395 19724918

[30] Zhang LY Zhang XC Wang LD Zhang ZF Li PL. Increased expression of GRP94 protein is associated with decreased sensitivity to adriamycin in ovarian carcinoma cell lines. Clin Exp Obstet Gynecol (2008) 35:257–63. 19205439

[31] Garg AD Krysko DV Vandenabeele P Agostinis P. Hypericin-based photodynamic therapy induces surface exposure of damage-associated molecular patterns like HSP70 and calreticulin. Cancer Immunol Immunother (2012) 61:215–21. 10.1007/s00262-011-1184-2 22193987 PMC11029694

[32] Korbelik M Zhang W Merchant S. Involvement of damage-associated molecular patterns in tumor response to photodynamic therapy: surface expression of calreticulin and high-mobility group box-1 release. Cancer Immunol Immunother (2011) 60:1431–7. 10.1007/s00262-011-1047-x 21644033 PMC11028986

[33] Zamanian M Veerakumarasivam A Abdullah S Rosli R Calreticulin and cancer. Pathol Oncol Res (2013) 19:149–54 10.1007/s12253-012-9600-2 23392843

[34] Gold LI Eggleton P Sweetwyne MT Van Duyn LB Greives MR Naylor SM Calreticulin: non-endoplasmic reticulum functions in physiology and disease. FASEB J (2010) 24:665–83. 10.1096/fj.09-145482 19940256 PMC2830142

[35] Scaffidi P Misteli T Bianchi ME. Release of chromatin protein HMGB1 by necrotic cells triggers inflammation. Nature (2002) 418:191–5. 10.1038/nature00858 12110890

[36] Dumitriu IE Baruah P Valentinis B Voll RE Herrmann M Nawroth PP Release of high mobility group box 1 by dendritic cells controls T cell activation via the receptor for advanced glycation end products. J Immunol (2005) 174:7506–15. 10.4049/jimmunol.174.12.7506 15944249

[37] Wang H Bloom O Zhang M Vishnubhakat JM Ombrellino M Che J HMG-1 as a late mediator of endotoxin lethality in mice. Science (1999) 285:248–51. 10.1126/science.285.5425.248 10398600

[38] Tamura Y Hirohashi Y Kutomi G Nakanishi K Kamiguchi K Torigoe T Tumor-produced secreted form of binding of immunoglobulin protein elicits antigen-specific tumor immunity. J Immunol (2011) 186:4325–30. 10.4049/jimmunol.1004048 21339366

[39] Udono H Levey DL Srivastava PK. Cellular requirements for tumor-specific immunity elicited by heat shock proteins: tumor rejection antigen gp96 primes CD8+ T cells in vivo. Proc Natl Acad Sci U S A (1994) 91:3077–81. 10.1073/pnas.91.8.3077 7909157 PMC43518

[40] Bodman-Smith MD Fife MF Wythe H Corrigal VM Panayi GS Wedderburn LR Anti-BiP antibody levels in juvenile idiopathic arthritis (JIA). Rheumatology (2004) 43:1305–6 10.1093/rheumatology/keh186 15448213

[41] Eggleton P Reid KB Kishore U Sontheimer RD. Clinical relevance of calreticulin in systemic lupus erythematosus. Lupus (1997) 6:564–71. 10.1177/096120339700600703 9302659

[42] Kishore U Sontheimer RD Sastry KN Zappi EG Hughes GR Khamashta MA The systemic lupus erythematosus (SLE) disease autoantigen-calreticulin can inhibit C1q association with immune complexes. Clin Exp Immunol (1997) 108:181–90. 10.1046/j.1365-2249.1997.3761273.x 9158084 PMC1904655

[43] Tarr JM Winyard PG Ryan B Harries LW Haigh R Viner N Extracellular calreticulin is present in the joints of patients with rheumatoid arthritis and inhibits FasL (CD95L)-mediated apoptosis of T cells. Arthritis Rheum (2010) 62:2919–29. 10.1002/art.27602 20533543

[44] Weber CK Haslbeck M Englbrecht M Sehnert B Mielenz D Graef D Antibodies to the endoplasmic reticulum-resident chaperones calnexin, BiP and Grp94 in patients with rheumatoid arthritis and systemic lupus erythematosus. Rheumatology (2010) 49:2255–63 10.1093/rheumatology/keq272 20716673

[45] Ling S Cline EN Haug TS Fox DA Holoshitz J. Citrullinated calreticulin potentiates rheumatoid arthritis shared epitope signaling. Arthritis Rheum (2013) 65:618–26. 10.1002/art.37814 23233327 PMC3582785

[46] Ling S Lai A Borschukova O Pumpens P Holoshitz J. Activation of nitric oxide signaling by the rheumatoid arthritis shared epitope. Arthritis Rheum (2006) 54:3423–32. 10.1002/art.22178 17075829

[47] Basu S Binder RJ Ramalingam T Srivastava PK. CD91 is a common receptor for heat shock proteins gp96, hsp90, hsp70, and calreticulin. Immunity (2001) 14:303–13. 10.1016/S1074-7613(01)00111-X 11290339

[48] Srivastava P. Interaction of heat shock proteins with peptides and antigen presenting cells: chaperoning of the innate and adaptive immune responses. Annu Rev Immunol (2002) 20:395–425. 10.1146/annurev.immunol.20.100301.064801 11861608

[49] Berwin B Reed RC Nicchitta CV. Virally induced lytic cell death elicits the release of immunogenic GRP94/gp96. J Biol Chem (2001) 276:21083–8. 10.1074/jbc.M101836200 11279246

[50] Garg AD Krysko DV Verfaillie T Kaczmarek A Ferreira GB Marysael T A novel pathway combining calreticulin exposure and ATP secretion in immunogenic cancer cell death. EMBO J (2012) 31:1062–79. 10.1038/emboj.2011.497 22252128 PMC3298003

[51] Clark PR Menoret A. The inducible Hsp70 as a marker of tumor immunogenicity. Cell Stress Chaperones (2001) 6:121–5. 10.1379/1466-1268(2001)006<0121:TIHAAM>2.0.CO;2 11599573 PMC434389

[52] Feng H Zeng Y Whitesell L Katsanis E. Stressed apoptotic tumor cells express heat shock proteins and elicit tumor-specific immunity. Blood (2001) 97:3505–12. 10.1182/blood.V97.11.3505 11369644

[53] Feng H Zeng Y Graner MW Katsanis E. Stressed apoptotic tumor cells stimulate dendritic cells and induce specific cytotoxic T cells. Blood (2002) 100:4108–15. 10.1182/blood-2002-05-1389 12393401

[54] Feng H Zeng Y Graner MW Likhacheva A Katsanis E. Exogenous stress proteins enhance the immunogenicity of apoptotic tumor cells and stimulate antitumor immunity. Blood (2003) 101:245–52. 10.1182/blood-2002-05-1580 12393411

[55] Obeid M Tesniere A Ghiringhelli F Fimia GM Apetoh L Perfettini JL Calreticulin exposure dictates the immunogenicity of cancer cell death. Nat Med (2007) 13:54–61. 10.1038/nm1523 17187072

[56] Vandivier RW Ogden CA Fadok VA Hoffmann PR Brown KK Botto M Role of surfactant proteins A, D, and C1q in the clearance of apoptotic cells in vivo and in vitro: calreticulin and CD91 as a common collectin receptor complex. J Immunol (2002) 169:3978–86. 10.4049/jimmunol.169.7.3978 12244199

[57] Donnelly S Roake W Brown S Young P Naik H Wordsworth P Impaired recognition of apoptotic neutrophils by the C1q/calreticulin and CD91 pathway in systemic lupus erythematosus. Arthritis Rheum (2006) 54:1543–56. 10.1002/art.21783 16645988

[58] Chao MP Jaiswal S Weissman-Tsukamoto R Alizadeh AA Gentles AJ Volkmer J Calreticulin is the dominant pro-phagocytic signal on multiple human cancers and is counterbalanced by CD47. Sci Transl Med (2010) 2:63ra94. 10.1126/scitranslmed.3001375 21178137 PMC4126904

[59] Chao MP Alizadeh AA Tang C Myklebust JH Varghese B Gill S Anti-CD47 antibody synergizes with rituximab to promote phagocytosis and eradicate non-Hodgkin lymphoma. Cell (2010) 142:699–713. 10.1016/j.cell.2010.07.044 20813259 PMC2943345

[60] Gardai SJ McPhillips KA Frasch SC Janssen WJ Starefeldt A Murphy-Ullrich JE Cell-surface calreticulin initiates clearance of viable or apoptotic cells through trans-activation of LRP on the phagocyte. Cell (2005) 123:321–34. 10.1016/j.cell.2005.08.032 16239148

[61] Kepp O Gdoura A Martins I Panaretakis T Schlemmer F Tesniere A Lysyl tRNA synthetase is required for the translocation of calreticulin to the cell surface in immunogenic death. Cell Cycle (2010) 9:3072–7. 10.4161/cc.9.15.12459 20699648

[62] Obeid M. ERP57 membrane translocation dictates the immunogenicity of tumor cell death by controlling the membrane translocation of calreticulin. J Immunol (2008) 181:2533–43. 10.4049/jimmunol.181.4.2533 18684944

[63] Ni M Wei W Wang Y Zhang N Ding H Shen C Serum levels of calreticulin in correlation with disease activity in patients with rheumatoid arthritis. J Clin Immunol (2013) 33:947–53. 10.1007/s10875-013-9885-2 23532497

[64] Kinoshita G Purcell AW Keech CL Farris AD McCluskey J Gordon TP. Molecular chaperones are targets of autoimmunity in Ro(SS-A) immune mice. Clin Exp Immunol (1999) 115:268–74. 10.1046/j.1365-2249.1999.00794.x 9933452 PMC1905168

[65] Casciola-Rosen LA Anhalt G Rosen A. Autoantigens targeted in systemic lupus erythematosus are clustered in two populations of surface structures on apoptotic keratinocytes. J Exp Med (1994) 179:1317–30. 10.1084/jem.179.4.1317 7511686 PMC2191465

[66] Matzinger P. Tolerance, danger, and the extended family. Annu Rev Immunol (1994) 12:991–1045. 10.1146/annurev.immunol.12.1.991 8011301

[67] Komurasaki R Imaoka S Tada N Okada K Nishiguchi S Funae Y. LKM-1 sera from autoimmune hepatitis patients that recognize ERp57, carboxylesterase 1 and CYP2D6. Drug Metab Pharmacokinet (2010) 25:84–92. 10.2133/dmpk.25.84 20208391

[68] Watanabe K Ohira H Orikasa H Saito K Kanno K Shioya Y Anti-calreticulin antibodies in patients with inflammatory bowel disease. Fukushima J Med Sci (2006) 52:1–11. 10.5387/fms.52.1 16995349

[69] Suzuki S Utsugisawa K Iwasa K Satoh T Nagane Y Yoshikawa H Autoimmunity to endoplasmic reticulum chaperone GRP94 in myasthenia gravis. J Neuroimmunol (2011) 237:87–92. 10.1016/j.jneuroim.2011.06.011 21774995

[70] Kreisel W Siegel A Bahler A Spamer C Schiltz E Kist M High prevalence of antibodies to calreticulin of the IgA class in primary biliary cirrhosis: a possible role of gut-derived bacterial antigens in its aetiology? Scand J Gastroenterol (1999) 34:623–8. 10.1080/003655299750026100 10440614

[71] Goeb V Thomas-L’Otellier M Daveau R Charlionet R Fardellone P Le Loet X Candidate autoantigens identified by mass spectrometry in early rheumatoid arthritis are chaperones and citrullinated glycolytic enzymes. Arthritis Res Ther (2009) 11:R38. 10.1186/ar2644 19284558 PMC2688184

[72] Eggleton P Ward FJ Johnson S Khamashta MA Hughes GR Hajela VA Fine specificity of autoantibodies to calreticulin: epitope mapping and characterization. Clin Exp Immunol (2000) 120:384–91. 10.1046/j.1365-2249.2000.01214.x 10792392 PMC1905652

[73] Nagayama S Yokoi T Tanaka H Kawaguchi Y Shirasaka T Kamataki T. Occurrence of autoantibody to protein disulfide isomerase in patients with hepatic disorder. J Toxicol Sci (1994) 19:163–9. 10.2131/jts.19.3_155 7966454

[74] Raiter A Vilkin A Gingold R Levi Z Halpern M Niv Y The presence of anti-GRP78 antibodies in the serum of patients with colorectal carcinoma: a potential biomarker for early cancer detection. Int J Biol Markers (2014) 29:e431–5. 10.5301/jbm.5000086 24803280

[75] Sanchez D Palova-Jelinkova L Felsberg J Simsova M Pekarikova A Pecharova B Anti-calreticulin immunoglobulin A (IgA) antibodies in refractory coeliac disease. Clin Exp Immunol (2008) 153:351–9. 10.1111/j.1365-2249.2008.03701.x 18637103 PMC2527357

[76] Hong SH Misek DE Wang H Puravs E Giordano TJ Greenson JK An autoantibody-mediated immune response to calreticulin isoforms in pancreatic cancer. Cancer Res (2004) 64:5504–10. 10.1158/0008-5472.CAN-04-0077 15289361

[77] Liu Y He J Xie X Su G Teitz-Tennenbaum S Sabel MS Serum autoantibody profiling using a natural glycoprotein microarray for the prognosis of early melanoma. J Proteome Res (2010) 9:6044–51. 10.1021/pr100856k 20879797 PMC2974814

[78] Platet N Cunat S Chalbos D Rochefort H Garcia M. Unliganded and liganded estrogen receptors protect against cancer invasion via different mechanisms. Mol Endocrinol (2000) 14:999–1009. 10.1210/mend.14.7.0492 10894150

[79] Arnaudeau S Frieden M Nakamura K Castelbou C Michalak M Demaurex N. Calreticulin differentially modulates calcium uptake and release in the endoplasmic reticulum and mitochondria. J Biol Chem (2002) 277:46696–705. 10.1074/jbc.M202395200 12324449

[80] Chen CN Chang CC Su TE Hsu WM Jeng YM Ho MC Identification of calreticulin as a prognosis marker and angiogenic regulator in human gastric cancer. Ann Surg Oncol (2009) 16:524–33. 10.1245/s10434-008-0243-1 19050968

[81] Lu YC Chen CN Wang B Hsu WM Chen ST Chang KJ Changes in tumor growth and metastatic capacities of J82 human bladder cancer cells suppressed by down-regulation of calreticulin expression. Am J Pathol (2011) 179:1425–33. 10.1016/j.ajpath.2011.05.015 21723245 PMC3157280

[82] Kageyama S Isono T Iwaki H Wakabayashi Y Okada Y Kontani K Identification by proteomic analysis of calreticulin as a marker for bladder cancer and evaluation of the diagnostic accuracy of its detection in urine. Clin Chem (2004) 50:857–66. 10.1373/clinchem.2003.027425 14764641

[83] Liu R Gong J Chen J Li Q Song C Zhang J Calreticulin as a potential diagnostic biomarker for lung cancer. Cancer Immunol Immunother (2012) 61:855–64. 10.1007/s00262-011-1146-8 22083347 PMC11029700

[84] Song MN Moon PG Lee JE Na M Kang W Chae YS Proteomic analysis of breast cancer tissues to identify biomarker candidates by gel-assisted digestion and label-free quantification methods using LC-MS/MS. Arch Pharm Res (2012) 35:1839–47. 10.1007/s12272-012-1018-6 23139137

[85] Galazis N Olaleye O Haoula Z Layfield R Atiomo W. Proteomic biomarkers for ovarian cancer risk in women with polycystic ovary syndrome: a systematic review and biomarker database integration. Fertil Steril (2012) 98:1590.e–601.e. 10.1016/j.fertnstert.2012.08.002 22959458

[86] Morelli M Scumaci D Di Cello A Venturella R Donato G Faniello MC DJ-1 in endometrial cancer: a possible biomarker to improve differential diagnosis between subtypes. Int J Gynecol Cancer (2014) 24:649–58. 10.1097/IGC.0000000000000102 24614826

[87] Yoon GS Lee H Jung Y Yu E Moon HB Song K Nuclear matrix of calreticulin in hepatocellular carcinoma. Cancer Res (2000) 60:1117–20. 10706133

[88] Alaiya A Roblick U Egevad L Carlsson A Franzen B Volz D Polypeptide expression in prostate hyperplasia and prostate adenocarcinoma. Anal Cell Pathol (2000) 21:1–9. 10.1155/2000/351963 11254220 PMC4618420

[89] Alur M Nguyen MM Eggener SE Jiang F Dadras SS Stern J Suppressive roles of calreticulin in prostate cancer growth and metastasis. Am J Pathol (2009) 175:882–90. 10.2353/ajpath.2009.080417 19608864 PMC2716982

[90] Liu X Ji J Forsti A Sundquist K Sundquist J Hemminki K Autoimmune disease and subsequent urological cancer. J Urol (2013) 189:2262–8 10.1016/j.juro.2012.12.014 23228387

[91] Hemminki K Liu X Forsti A Ji J Sundquist J Sundquist K. Subsequent leukaemia in autoimmune disease patients. Br J Haematol (2013) 161:677–87. 10.1111/bjh.12330 23565673

[92] Hemminki K Liu X Forsti A Ji J Sundquist J Sundquist K. Subsequent brain tumors in patients with autoimmune disease. Neuro Oncol (2013) 15:1142–50. 10.1093/neuonc/not070 23757294 PMC3748918

[93] Castro FA Liu X Forsti A Ji J Sundquist J Sundquist K Increased risk of hepatobiliary cancers after hospitalization for autoimmune disease. Clin Gastroenterol Hepatol (2014) 12:1038–45.e7. 10.1016/j.cgh.2013.11.007 24246767

[94] Aguilar-Guzman L Lobos-Gonzalez L Rosas C Vallejos G Falcon C Sosoniuk E Human survivin and *Trypanosoma cruzi* calreticulin act in synergy against a murine melanoma in vivo. PLoS One (2014) 9:e95457. 10.1371/journal.pone.0095457 24755644 PMC3995754

[95] Ramirez G Valck C Aguilar L Kemmerling U Lopez-Munoz R Cabrera G Roles of *Trypanosoma cruzi* calreticulin in parasite-host interactions and in tumor growth. Mol Immunol (2012) 52:133–40. 10.1016/j.molimm.2012.05.006 22673211

[96] Lopez NC Valck C Ramirez G Rodriguez M Ribeiro C Orellana J Antiangiogenic and antitumor effects of *Trypanosoma cruzi* calreticulin. PLoS Negl Trop Dis (2010) 4:e730. 10.1371/journal.pntd.0000730 20625551 PMC2897838

[97] Munro S Pelham HR. A C-terminal signal prevents secretion of luminal ER proteins. Cell (1987) 48:899–907. 10.1016/0092-8674(87)90086-9 3545499

[98] Altmeyer A Maki RG Feldweg AM Heike M Protopopov VP Masur SK Tumor-specific cell surface expression of the-KDEL containing, endoplasmic reticular heat shock protein gp96. Int J Cancer (1996) 69:340–9. 10.1002/(SICI)1097-0215(19960822)69:4<340::AID-IJC18>3.0.CO;2-9 8797880

[99] Robert J Menoret A Cohen N. Cell surface expression of the endoplasmic reticular heat shock protein gp96 is phylogenetically conserved. J Immunol (1999) 163:4133–9. 10510348

[100] Wiest DL Bhandoola A Punt J Kreibich G McKean D Singer A. Incomplete endoplasmic reticulum (ER) retention in immature thymocytes as revealed by surface expression of “ER-resident” molecular chaperones. Proc Natl Acad Sci U S A (1997) 94:1884–9. 10.1073/pnas.94.5.1884 9050874 PMC20012

[101] Sauk JJ Norris K Hebert C Ordonez J Reynolds M. Hsp47 binds to the KDEL receptor and cell surface expression is modulated by cytoplasmic and endosomal pH. Connect Tissue Res (1998) 37:105–19. 10.3109/03008209809028904 9643651

[102] Nangalia J Massie CE Baxter EJ Nice FL Gundem G Wedge DC Somatic CALR mutations in myeloproliferative neoplasms with nonmutated JAK2. N Engl J Med (2013) 369:2391–405. 10.1056/NEJMoa1312542 24325359 PMC3966280

[103] Vannucchi AM Rotunno G Bartalucci N Raugei G Carrai V Balliu M Calreticulin mutation-specific immunostaining in myeloproliferative neoplasms: pathogenetic insight and diagnostic value. Leukemia (2014) 28:1811–8. 10.1038/leu.2014.100 24618731 PMC4158831

[104] Capitani M Sallese M. The KDEL receptor: new functions for an old protein. FEBS Lett (2009) 583:3863–71. 10.1016/j.febslet.2009.10.053 19854180

[105] Cancino J Jung JE Luini A. Regulation of Golgi signaling and trafficking by the KDEL receptor. Histochem Cell Biol (2013) 140:395–405. 10.1007/s00418-013-1130-9 23873287

[106] Han JM Park SG Liu B Park BJ Kim JY Jin CH Aminoacyl-tRNA synthetase-interacting multifunctional protein 1/p43 controls endoplasmic reticulum retention of heat shock protein gp96: its pathological implications in lupus-like autoimmune diseases. Am J Pathol (2007) 170:2042–54. 10.2353/ajpath.2007.061266 17525271 PMC1899434

[107] Townsley FM Wilson DW Pelham HR. Mutational analysis of the human KDEL receptor: distinct structural requirements for Golgi retention, ligand binding and retrograde transport. EMBO J (1993) 12:2821–9. 8392934 10.1002/j.1460-2075.1993.tb05943.x PMC413532

[108] Pulvirenti T Giannotta M Capestrano M Capitani M Pisanu A Polishchuk RS A traffic-activated Golgi-based signalling circuit coordinates the secretory pathway. Nat Cell Biol (2008) 10:912–22. 10.1038/ncb1751 18641641

[109] Giannotta M Ruggiero C Grossi M Cancino J Capitani M Pulvirenti T The KDEL receptor couples to Galphaq/11 to activate Src kinases and regulate transport through the Golgi. EMBO J (2012) 31:2869–81. 10.1038/emboj.2012.134 22580821 PMC3395092

[110] Panaretakis T Kepp O Brockmeier U Tesniere A Bjorklund AC Chapman DC Mechanisms of pre-apoptotic calreticulin exposure in immunogenic cell death. EMBO J (2009) 28:578–90. 10.1038/emboj.2009.1 19165151 PMC2657583

[111] Denning GM Leidal KG Holst VA Iyer SS Pearson DW Clark JR Calreticulin biosynthesis and processing in human myeloid cells: demonstration of signal peptide cleavage and N-glycosylation. Blood (1997) 90:372–81. 9207473

[112] Tarr JM Young PJ Morse R Shaw DJ Haigh R Petrov PG A mechanism of release of calreticulin from cells during apoptosis. J Mol Biol (2010) 401:799–812. 10.1016/j.jmb.2010.06.064 20624402

[113] Tsai B Ye Y Rapoport TA. Retro-translocation of proteins from the endoplasmic reticulum into the cytosol. Nat Rev Mol Cell Biol (2002) 3:246–55. 10.1038/nrm780 11994744

[114] Afshar N Black BE Paschal BM. Retrotranslocation of the chaperone calreticulin from the endoplasmic reticulum lumen to the cytosol. Mol Cell Biol (2005) 25:8844–53. 10.1128/MCB.25.20.8844-8853.2005 16199864 PMC1265792

[115] Holaska JM Black BE Love DC Hanover JA Leszyk J Paschal BM Calreticulin is a receptor for nuclear export. J Cell Biol (2001) 152:127–40 10.1083/jcb.152.1.127 11149926 PMC2193655

[116] Lopez Sambrooks C Carpio MA Hallak ME. Arginylated calreticulin at plasma membrane increases susceptibility of cells to apoptosis. J Biol Chem (2012) 287:22043–54. 10.1074/jbc.M111.338335 22577148 PMC3381163

[117] Saha S Kashina A Posttranslational arginylation as a global biological regulator. Dev Biol (2011) 358:1–8 10.1016/j.ydbio.2011.06.043 21784066 PMC3171647

[118] Wang J Han X Wong CC Cheng H Aslanian A Xu T Arginyltransferase ATE1 catalyzes midchain arginylation of proteins at side chain carboxylates in vivo. Chem Biol (2014) 21:331–7. 10.1016/j.chembiol.2013.12.017 24529990 PMC4010198

[119] Decca MB Carpio MA Bosc C Galiano MR Job D Andrieux A Post-translational arginylation of calreticulin: a new isospecies of calreticulin component of stress granules. J Biol Chem (2007) 282:8237–45. 10.1074/jbc.M608559200 17197444 PMC2702537

[120] Ling S Cheng A Pumpens P Michalak M Holoshitz J. Identification of the rheumatoid arthritis shared epitope binding site on calreticulin. PLoS One (2010) 5:e11703. 10.1371/journal.pone.0011703 20661469 PMC2908537

[121] Ling S Pi X Holoshitz J. The rheumatoid arthritis shared epitope triggers innate immune signaling via cell surface calreticulin. J Immunol (2007) 179:6359–67. 10.4049/jimmunol.179.9.6359 17947714

[122] Multhoff G Hightower LE Cell surface expression of heat shock proteins and the immune response. Cell Stress Chaperones (1996) 1:167–76 10.1379/1466-1268(1996)001<0167:CSEOHS>2.3.CO;2 9222602 PMC248476

[123] Arispe N De Maio A. ATP and ADP modulate a cation channel formed by Hsc70 in acidic phospholipid membranes. J Biol Chem (2000) 275:30839–43. 10.1074/jbc.M005226200 10899168

[124] Paidassi H Tacnet-Delorme P Verneret M Gaboriaud C Houen G Duus K Investigations on the C1q-calreticulin-phosphatidylserine interactions yield new insights into apoptotic cell recognition. J Mol Biol (2011) 408:277–90. 10.1016/j.jmb.2011.02.029 21352829

[125] Oyadomari S Takeda K Takiguchi M Gotoh T Matsumoto M Wada I Nitric oxide-induced apoptosis in pancreatic beta cells is mediated by the endoplasmic reticulum stress pathway. Proc Natl Acad Sci U S A (2001) 98:10845–50. 10.1073/pnas.191207498 11526215 PMC58562

[126] Patel JM Li YD Zhang J Gelband CH Raizada MK Block ER. Increased expression of calreticulin is linked to ANG IV-mediated activation of lung endothelial NOS. Am J Physiol (1999) 277:L794–801. 10516221 10.1152/ajplung.1999.277.4.L794

[127] Apetoh L Ghiringhelli F Tesniere A Obeid M Ortiz C Criollo A Toll-like receptor 4-dependent contribution of the immune system to anticancer chemotherapy and radiotherapy. Nat Med (2007) 13:1050–9 10.1038/nm1622 17704786

[128] Ghiringhelli F Apetoh L Tesniere A Aymeric L Ma Y Ortiz C Activation of the NLRP3 inflammasome in dendritic cells induces IL-1beta-dependent adaptive immunity against tumors. Nat Med (2009) 15:1170–8. 10.1038/nm.2028 19767732

[129] D’Eliseo D Manzi L Velotti F. Capsaicin as an inducer of damage-associated molecular patterns (DAMPs) of immunogenic cell death (ICD) in human bladder cancer cells. Cell Stress Chaperones (2013) 18:801–8. 10.1007/s12192-013-0422-2 23580156 PMC3789874

[130] Peters LR Raghavan M. Endoplasmic reticulum calcium depletion impacts chaperone secretion, innate immunity, and phagocytic uptake of cells. J Immunol (2011) 187:919–31. 10.4049/jimmunol.1100690 21670312 PMC3371385

[131] Wemeau M Kepp O Tesniere A Panaretakis T Flament C De Botton S Calreticulin exposure on malignant blasts predicts a cellular anticancer immune response in patients with acute myeloid leukemia. Cell Death Dis (2010) 1:e104. 10.1038/cddis.2010.82 21368877 PMC3032293

[132] Kepp O Galluzzi L Giordanetto F Tesniere A Vitale I Martins I Disruption of the PP1/GADD34 complex induces calreticulin exposure. Cell Cycle (2009) 8:3971–7. 10.4161/cc.8.23.10191 19901557

[133] Martins I Kepp O Schlemmer F Adjemian S Tailler M Shen S Restoration of the immunogenicity of cisplatin-induced cancer cell death by endoplasmic reticulum stress. Oncogene (2011) 30:1147–58. 10.1038/onc.2010.500 21151176

[134] Tufi R Panaretakis T Bianchi K Criollo A Fazi B Di Sano F Reduction of endoplasmic reticulum Ca2+ levels favors plasma membrane surface exposure of calreticulin. Cell Death Differ (2008) 15:274–82. 10.1038/sj.cdd.4402275 18034188

[135] Zappasodi R Pupa SM Ghedini GC Bongarzone I Magni M Cabras AD Improved clinical outcome in indolent B-cell lymphoma patients vaccinated with autologous tumor cells experiencing immunogenic death. Cancer Res (2010) 70:9062–72. 10.1158/0008-5472.CAN-10-1825 20884630

[136] Fucikova J Kralikova P Fialova A Brtnicky T Rob L Bartunkova J Human tumor cells killed by anthracyclines induce a tumor-specific immune response. Cancer Res (2011) 71:4821–33. 10.1158/0008-5472.CAN-11-0950 21602432

[137] Menger L Vacchelli E Adjemian S Martins I Ma Y Shen S Cardiac glycosides exert anticancer effects by inducing immunogenic cell death. Sci Transl Med (2012) 4:143ra99. 10.1126/scitranslmed.3003807 22814852

[138] Fredly H Ersvaer E Gjertsen BT Bruserud O. Immunogenic apoptosis in human acute myeloid leukemia (AML): primary human AML cells expose calreticulin and release heat shock protein (HSP) 70 and HSP90 during apoptosis. Oncol Rep (2011) 25:1549–56. 10.3892/or.2011.1229 21431284

[139] Jeffery E Peters LR Raghavan M. The polypeptide binding conformation of calreticulin facilitates its cell-surface expression under conditions of endoplasmic reticulum stress. J Biol Chem (2011) 286:2402–15. 10.1074/jbc.M110.180877 21075854 PMC3024734

